# Corneal Endothelial Morphology and Thickness Changes in Patients with Gout

**DOI:** 10.4274/tjo.galenos.2018.01947

**Published:** 2019-09-03

**Authors:** Pınar Kösekahya, Cemile Üçgül Atılgan, Kadir Gökhan Atılgan, Mustafa Koç, Kemal Tekin, Mehtap Çağlayan, Yasin Şakir Göker

**Affiliations:** 1 Ulucanlar Eye Training and Research Hospital, Ophthalmology Clinic, Ankara, Turkey; 2 Dışkapı Training and Research Hospital, Nephrology Clinic, Ankara, Turkey; 3 Van Erciş State Hospital, Ophthalmology Clinic, Van, Turkey; 4 Gazi Yaşargil Training and Research Hospital, Ophthalmology Clinic, Diyarbakır, Turkey

**Keywords:** Gout, corneal endothelial cell density, corneal endothelial function, specular microscopy

## Abstract

**Objectives:**

To investigate corneal endothelial cell density (ECD), morphology, and central corneal thickness (CCT) in patients with gout compared with healthy subjects.

**Materials and Methods:**

Fifty eyes of 50 gout patients and 50 eyes of 50 healthy subjects without gout or any other systemic disease were included in this study. After detailed ophthalmologic examination, specular microscopy (Tomey EM-4000; Tomey Corp) measurement was performed for all participants. ECD, average cell area (ACA), coefficient of variation (CV), hexagonality ratio, and CCT values were recorded.

**Results:**

Mean ECD and hexagonality ratio were lower (p=0.004 and p=0.002) and CV, ACA, and CCT values were higher (p=0.001, p=0.007, and p=0.001) in patients with gout when compared to healthy subjects. There were significant correlations between gout disease duration and CD and hexagonality ratio (p=0.019 and p=0.043) and also between uric acid value and hexagonality ratio and CCT (p=0.044 and p=0.003).

**Conclusion:**

Altered corneal endothelial function was found in patients with gout when compared to healthy subjects and the alteration increased as gout duration and uric acid value increased.

## Introduction

Gout is an inflammatory metabolic disorder characterized by the accumulation of monosodium urate (MSU) in the extracellular spaces, especially joints.^1^ High uric acid levels (≥6.8 mg/dL) is the most important risk factor, and the condition is triggered by the precipitation of MSU crystals from oversaturated body fluid into the ligaments, soft tissues, and intraarticular space.^2^ Its clinical characteristics include arthritis attacks, nephrolithiasis, nephropathy, and tophi.^3^

The global prevalence of gout has been reported as 1-4%.^4^ In two prevalence studies conducted in Turkey, the prevalence of gout was found to be 0.02% in the Havsa region and 0.31% in the İzmir region.^5,6^ Although the frequency of gout varies between societies due to genetic and cultural differences^7^, its prevalence worldwide nearly doubled between 1990 and 2010.^8^ This increase may be related to changes in eating habits and increasing obesity rates. There was a 3-fold increase in the number of academic studies on gout between 2005 and 2015.^9^

Ocular tophi have been demonstrated in the lateral canthus, upper eyelid, orbit, iris, anterior chamber, subconjunctival space, and cornea.^10^ Uric acid deposition in the cornea was confirmed by polarized light microscopy in two case reports.^11,12^

Good visual acuity requires a transparent cornea, and a transparent cornea requires healthy endothelial function and healthy stromal layer organization.^13^ Measurement of corneal transparency can be done using corneal densitometry analysis. In one of our previous studies, we evaluated corneal densitometry in gout patients and found increased corneal densitometry values indicating reduced corneal transparency in gout patients compared to healthy individuals.^14^ Tekin et al.^15^ demonstrated a correlation between corneal densitometry analysis and corneal endothelial morphometry in healthy corneas and stated that corneal densitometry may be an indicator of endothelial health.

Reduced corneal transparency in gout patients may be a result of uric acid accumulation in the stroma or endothelial dysfunction. Therefore, in this study we aimed to investigate corneal endothelial cell density, morphology, and central corneal thickness in gout patients compared to healthy individuals.

## Materials and Methods

This prospective study was conducted in accordance with the Declaration of Helsinki and legal regulations. All patients and healthy participants signed a written informed consent form approved by the local ethics committee (1151/2017).

Patients with gout were included in the study based on 2015 gout classification criteria from the American College of Rheumatology.^16^ These criteria include clinical (joint/bursa involvement, characteristic symptomatic episodes), laboratory (serum uric acid levels, MSU crystals in the synovial fluid), and imaging findings.

Individuals with systemic diseases other than gout, such as diabetes mellitus, chronic kidney disease, heart disease, and cancer, and individuals using systemic drugs that may increase uric acid levels were excluded from the study. Individuals with history of intraocular surgery, trauma, uveitis, optic nerve disease, glaucoma, corneal scar and ectasia, contact lens use, or topical eye drop use were not included.

Fifty eyes of 50 patients followed for gout in the nephrology unit of the Dışkapı Training and Research Hospital and 50 eyes of 50 healthy individuals without gout or any other systemic diseases were included in the study. Disease duration and uric acid levels were recorded for the gout patients, all of whom were using low-dose colchicine (0.5-1.5 mg/day). Allopurinol was adjusted according to the patients’ uric acid levels.

All patients and healthy individuals underwent best corrected visual acuity examination with Snellen chart, slit-lamp biomicroscopy examination, and fundus examination. Measurements of all patients were performed by the same technician using a noncontact specular microscope (Tomey EM-4000; Tomey Corp). All measurements were performed at least 3 times using the “center” method and at least 110 cells were included in each measurement. Endothelial cell density (ECD), average cell area (ACA), minimum cell area (CAmin), maximum cell area (CAmax), (SD) standard deviation of cell area, coefficient of variation in cell area (CV), hexagonal cell ratio (HEX), and central corneal thickness (CCT) were noted. Intraocular pressure (IOP) was measured using Goldmann applanation tonometry and corrected according to the Ehlers formula (corrected IOP = uncorrected IOP - [CCT - 520] x [5/70]).

### Statistical Analysis

Although both eyes were examined, for statistical analysis one eye of each participant was selected by generating random numbers using Microsoft Excel software. Statistical analysis of the data was performed using SPSS 22.0 software (SPSS Inc., Chicago, Illinois, USA). Descriptive statistics are expressed as mean ± SD. Categorical variables were compared using chi-square test. Independent-samples t-test was used to compare two groups. Correlations between gout duration and uric acid levels and endothelial morphometry were analyzed using Pearson correlation test. A p value <0.05 was considered statistically significant.

## Results

Mean age was 59.12±7.50 years in the gout group and 59.92±7.45 years in the control group (p=0.59). There were 18 women and 32 men in the gout group and 27 women and 23 men in the control group (p=0.10).

The mean disease duration in the gout group was 79.05±62.39 (12–240) months. Mean uric acid level was 8.49±2.14 mg/dL in the gout group and 3.30±0.75 mg/dL in the control group (p<0.001).

Best corrected visual acuity values on Snellen chart were 0.89±0.11 in the gout group and 0.92±0.10 in the control group (p=0.15). Mean Goldmann IOP was 17.04±3.82 mmHg in the gout group and 15.05±3.33 mmHg in the control group (p=0.03), while mean corrected IOP values in the two groups were 14.44±2.57 mmHg and 14.26±2.60 mmHg, respectively (p=0.72).

The mean ECD and HEX values were lower in the gout group than in the control group (p=0.004 and p=0.002). CV, SD, ACA, CAmax, and CCT values were significantly higher in the gout group than in the control group (p=0.001, p<0.001, p=0.007, p=0.002 and p=0.001). CAmin was also higher in the gout group, but the difference was not statistically significant (p=0.176) (Table 1).

Gout duration was negatively correlated with ECD and HEX (r=-0.400, p=0.019 and r=-0.348, p=0.043, respectively). In addition, there was a significant negative correlation between uric acid level and HEX (r=-0.245, p=0.044), and a significant positive correlation between uric acid levels and CCT (r=0.355, p=0.003) (Table 2).

## Discussion

The corneal endothelium is a nonmitotic tissue that plays a crucial role in the maintenance of corneal transparency. Similar to vascular endothelial cells, the corneal endothelium acts as a barrier.^17^ It maintains the equilibrium between aqueous humor flow into the stroma and pumping of aqueous humor from the stroma to the anterior chamber. The age-related decrease in endothelial cell number in a healthy cornea is compensated by increased Na,K-ATPase activity, which is the basis of endothelial pump function.^18^ Increased endothelial permeability and inability of the metabolic pump to compensate for the influx leads to stromal thickening and initiates a process that later results in corneal edema.^19^

Specular microscopy is used for noninvasive imaging and morphological analysis of the corneal endothelium. ECD is the most commonly used parameter to determine endothelial function and is expressed as cell number per mm^2^.^20^ In addition to ECD, CV and HEX are often used to evaluate endothelial morphology and stability. CV is an indicator of variation in cell area and is an objective criterion of polymegathism. It is the ratio between the SD of ACA in the endothelial zone to ACA, and should ideally be below 30%. HEX is the ratio of hexagonal cells to those of other geometric shapes. Although the ideal ratio is 100%, it has been reported in the 60-70% range in studies of healthy patients.^21^ CCT can be used as a marker that provides information about both the barrier and pump functions of the endothelium, and can be measured using specular microscopy.^20^

In a study including 380 gout patients, crystal deposits were detected in the corneas of 2 patients, located in the corneal epithelium in 1 case and the corneal stroma in the other.^22^ In another study evaluating 69 gout patients, deposits were detected in the corneal stroma of 1 patient.^23^ To the best of our knowledge, there are no studies in the literature that investigate the corneal epithelium in gout patients. In our study, gout patients had lower ACA and HEX values and higher CV and CCT values compared to healthy controls. These findings indicate that corneal endothelial functions are poorer in gout patients compared to age- and sex-matched healthy individuals.

In humans, the gene for urease, which is responsible for uric acid catabolism, is nonfunctional; as a result, overproduction or reduced excretion of uric acid leads to hyperuricemia.^24^ The best known disease related to hyperuricemia is gout, although it has also been associated with systemic inflammation, cardiovascular diseases, hypertension, and endothelial dysfunction in the literature.^25,26,27^ Hyperuricemia was shown to induce oxidative stress and inflammatory response, thus reducing nitric oxide release and causing endothelial dysfunction.^28^ It was reported that ocular inflammation negatively affects corneal endothelial functions and increases corneal thickness, and in another study, corneal endothelial changes were even observed in Behçet’s patients without ocular involvement.^29,30^ Uric acid deposition in the anterior chamber has been previously demonstrated in individuals with gout.^31^ Uremic toxins can disrupt the balance between both proinflammatory and inflammatory factors and proapoptotic and apoptotic factors.^32^ Disruption of the apoptotic equilibrium may accelerate apoptosis in endothelial cells that are in contact with uric acid in the anterior chamber,^33^ and the higher apoptosis rate may explain the low ECD in gout patients.

While ECD and HEX decrease with longer duration of gout, HEX decreases and CCT increases with higher uric acid level. An in vitro study showed that Na,K-ATPase activity decreases in a dose-dependent manner in cells exposed to increasing doses of uric acid.^34^The longer a patient has been followed for gout and the higher their uric acid levels, the more corneal endothelial dysfunction they have. This shows the importance of close monitoring of uric acid levels in patients with gout.

Increased CCT is caused by corneal endothelial dysfunction and the subsequent increase in stromal hydration; increased stromal hydration is also important because it explains the decreased corneal transparency in gout patients shown in our previous study.^14^ Another importance of CCT is that it impacts IOP values measured using Goldmann applanation tonometry, which is still the gold standard in IOP measurement.^35^ IOP measurements obtained using this method may be incorrectly high in thick corneas and incorrectly low in thin corneas. In our study, Goldmann IOP was significantly higher in the gout group than the control group, whereas IOP values adjusted for CCT were similar in both groups. It might be useful to take the increase in CCT into consideration when interpreting IOP measurements in gout patients.

Colchicine is an alkaloid that exerts anti-inflammatory activity by disrupting intracellular microtubule and leukocyte function.^36^ Colchicine has been detected in the tears of patients using systemic colchicine.^37^ Because colchicine inhibits neutrophil migration and expression of adhesion molecules, it was claimed to delay the recovery of corneal epithelial damage.^38^ While there are no studies on its direct effect on corneal epithelial cells, it is presumed to affect disease activity and the amount of inflammatory mediators and cytokines. These anti-inflammatory properties are expected to protect against uric acid damage rather than reducing corneal endothelial function, but in vitro studies are required to corroborate these hypotheses.

### Study Limitations

A limitation of our study may be error caused by specular microscopic measurements. In order to minimize this error, all measurements were performed by the same technician and were repeated three times. Another limitation is the systemic drugs used by gout patients. All patients were using colchicine and allopurinol. There are no studies regarding whether these drugs affect the corneal endothelium. In vitro studies evaluating the effect of colchicine on the corneal endothelium may be beneficial. The effects of uric acid on the corneal epithelium should also be demonstrated with in vitro studies. Further research that includes laboratory analyses in larger patient series is needed.

## Conclusion

Our study showed that in patients with gout, corneal endothelial dysfunction tends to increase with disease duration and uncontrolled uric acid levels. Uric acid deposition may disrupt endothelial stability and increase corneal thickness by reducing Na,K-ATPase pump activity. The effects of chronic hyperuricemia on the corneal endothelium should be taken into consideration when examining gout patients for potential complications and treating diseases that increase with age, such as cataract and glaucoma.

## Figures and Tables

**Table 1:**
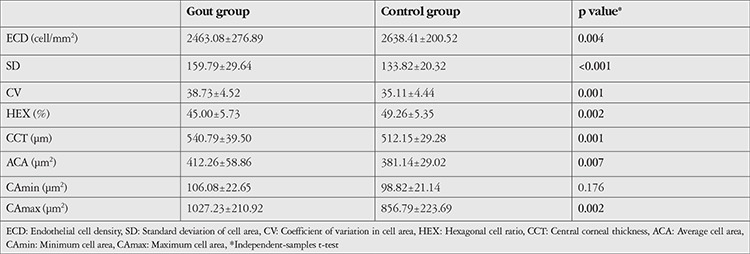
Comparison of corneal endothelial and thickness values in gout patients and healthy controls

**Table 2:**
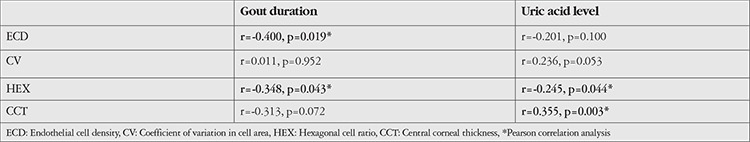
Analysis of correlations between corneal parameters and gout duration and uric acid levels
